# Hybrid Memetic Algorithm for the Node Location Problem in Local Positioning Systems

**DOI:** 10.3390/s20195475

**Published:** 2020-09-24

**Authors:** Javier Díez-González, Paula Verde, Rubén Ferrero-Guillén, Rubén Álvarez, Hilde Pérez

**Affiliations:** Department of Mechanical, Computer and Aerospace Engineering, Universidad de León, 24071 León, Spain; pgarcv00@estudiantes.unileon.es (P.V.); rferrg00@estudiantes.unileon.es (R.F.-G.); ruben.alvarez@drotium.com (R.Á.); hilde.perez@unileon.es (H.P.)

**Keywords:** clock errors, Cramér–Rao bound, genetic algorithm, hybrid genetic algorithm, local positioning systems, memetic algorithm, node location problem, noise uncertainties

## Abstract

Local Positioning Systems (LPS) have shown excellent performance for applications that demand high accuracy. They rely on ad-hoc node deployments which fit the environment characteristics in order to reduce the system uncertainties. The obtainment of competitive results through these systems requires the solution of the Node Location Problem (finding the optimal cartesian coordinates of the architecture sensors). This problem has been assigned as NP-Hard, therefore a heuristic solution is recommended for addressing this complex problem. Genetic Algorithms (GA) have shown an excellent trade-off between diversification and intensification in the literature. However, in Non-Line-of-Sight (NLOS) environments in which there is not continuity in the fitness function evaluation of a particular node distribution among contiguous solutions, challenges arise for the GA during the exploration of new potential regions of the space of solutions. Consequently, in this paper, we first propose a Hybrid GA with a combination of the GA operators in the evolutionary process for the Node Location Problem. Later, we introduce a Memetic Algorithm (MA) with a Local Search (LS) strategy for exploring the most different individuals of the population in search of improving the previous results. Finally, we combine the Hybrid Genetic Algorithm (HGA) and Memetic Algorithm (MA), designing an enhanced novel methodology for solving the Node Location Problem, a Hybrid Memetic Algorithm (HMA). Results show that the HMA proposed in this article outperforms all of the individual configurations presented and attains an improvement of 14.2% in accuracy for the Node Location Problem solution in the scenario of simulations with regards to the previous GA optimizations of the literature.

## 1. Introduction

The definition of the location of a target is an essential fact for performing complex tasks. Traditionally, Global Navigation Satellite Systems (GNSS) have been used for providing a stable signal for many different applications such as navigation, earth observation, emergency and rescue operations or surveillance. However, their signals are notably affected in their paths from satellites to targets and the accuracy achieved by these systems can be compromised by ionospheric instabilities [1], synchronization effects among the system devices [2], multipath phenomena [3] or signal path noise degradation [4].

However, the uncertainty in the position determination using GNSS may preclude their usage for applications that demand high accuracy (e.g., autonomous navigation, indoor localization, low-level UAV flights or precision agriculture). Therefore, new localization schemes based on the terrestrial deployment of sensors with target proximity are collecting notable research interest over the last few years [5,6]. These deployments, known as Local Positioning Systems (LPS), require an ad-hoc distribution of the sensors in space, adapting the sensor location to the characteristics of the environment of operation, thus reducing the system uncertainties in the position determination. The knowledge of the environment and the optimal deployment of the sensors enables mitigating or avoiding the main system error sources, thus producing competitive and cost-effective systems for applications that demand high accuracy [7].

LPS are categorized through the physical property measured for calculating the target location: power [8], angle [9], phase [10], frequency [11], time [12] and hybridizations of them [13,14].

Among them, time-based positioning (TBP) shows the best trade-off considering accuracy, reliability, robustness, stability and easy-to-implement hardware configurations. TBP is based on the measurement of the positioning signal time travel from an emitter to a receiver. There exist different architectures for computing the time measurements which produces different target determination calculations.

Time of Arrival (TOA) architectures measure the total time-of-flight of the positioning signal from an emitter to a receiver [15]. It requires the synchronism of the clock of all the system elements since every reception of the signal produces a different equation of a sphere for the target location determination. Generally, 3D positioning needs 4 receivers to unequivocally determine the target spatial coordinates. Time Difference of Arrival (TDOA) architecture measures the relative time lapse among the reception of the positioning signal in two different receivers [16]. Relative-time measurements generate hyperboloid surfaces of possible target locations in 3D. The necessity of using two different receivers for obtaining a hyperboloid equation means that 5 sensors are required to unequivocally determine the target location. However, we have proven [17] that under optimized sensor distributions this problem may be solved with 4 receivers.

TDOA architectures do not require the synchronism of the target with the system clocks. Even completely asynchronous architectures are recently being proposed [18,19] and are attracting high research interest since they collect all the time measurements in a single clock of a Coordinator Sensor (CS). This allows us to avoid synchronism among the system receivers, consequently reducing the architecture clock errors [20] but increasing the signal paths and noise errors [21], since they rely on a receive-and-retransmit strategy of the positioning signals in the Target Sensor (TS), producing longer path signals. In addition, a possible CS malfunction may produce temporal system unavailability [22] due to the particular architecture dependence on the CS.

These facts make the usage of synchronous and asynchronous TDOA deployments dependent on the environment characteristics. In this paper, we will analyze the asynchronous TDOA architecture since it supposes a promising technology in LPS that requires the solution of the Node Location Problem (NLP) to any application scenario and due to the extent usage of TDOA positioning in terrestrial localization [23,24].

However, regardless of the architecture used in local TBP, the optimal performance of the positioning system is achieved through the minimization of the system uncertainties in every possible TS location. This optimization demands an enhanced node distribution (i.e., an ad-hoc sensor location for the operating environment) in which noise uncertainties in Line-of-Sight (LOS) and Non-Line-of-Sight (NLOS) conditions and clock errors are reduced, favoring the optimal performance of the LPS.

The uncertainties are usually modeled in the Cramér–Rao Lower Bound (CRLB), since it provides the minimum achievable error by any positioning system in a defined TS location [25] and its usage is widespread in the field [26,27,28]. Kaune et al. [25] provided a matrix form of the Fisher Information Matrix (FIM), which is a maximum likelihood estimator for which the inverse is the CRLB. This FIM matrix includes the covariance matrix of the system in which the definition of the uncertainties is introduced.

The signal path noise degradation must deal with heteroscedastic noises [29], since the signal paths notably differ among sensors in LPS. For the characterization of the clock errors, we have recently introduced [20] a model in which the initial-time offset, clock drift and the instrument truncating error are considered. The minimization of this combined CRLB model enables the optimal performance of any LPS architecture for a defined TS location.

The finding of an optimized sensor location for high-demanded LPS applications, known as the Node Location Problem, must assume the overall minimization of the CRLB for each possible TS location, the Target Location Environment (TLE) [30]. This process is not derivable for all the TLE jointly [27,31] and it has been categorized as NP-Hard [32,33].

Therefore, a heuristic solution is recommended for addressing the optimization. Many different metaheuristic techniques such as simulated annealing [34], dolphin swarm algorithm [35], bat algorithm [36], elephant herding optimization [37] or diversified local search [38] have been used for approaching the node location problem, but especially Genetic Algorithms (GA) have been used in the node location problem [39,40,41] due to the excellent trade-off of the GA between diversification (i.e., the capacity to explore the space of solutions) and intensification (i.e., the finding of the optimal solution in a reduced part of the space of solutions) [42].

In our previous research, we have applied GA to the NLP in LPS [7,20,21,22,30]. In these papers, we have observed that the dimensions of the space of solutions which increases with the number of sensors, the resolution of the pre-defined possible space locations for them and the complexity of the fitness function evaluation, significantly affect the stable performance of the GA. This is due to the difficulty of exploring the huge space of solutions generated in the NLP.

In addition, the analysis of contiguous solutions (i.e., node distributions that differ only in a cartesian coordinate of a particular node) may suppose notable changes in the fitness function evaluation. These conditions especially occur in NLOS scenarios in which the signal quality may be significantly distorted if a node is located just behind an obstacle. This fact has promoted the usage of pre-defined populations of the GA for obtaining practical results in the GA evolution [21].

Thus, the observance of these facts has shown the necessity of introducing some knowledge in the optimization process of the node location problem in localization. In this paper, we first address the problem by constructing a Hybrid Genetic Algorithm (HGA) affecting the diversification and intensification phases through an ad-hoc usage of the GA operators for favoring the obtainment of practical results.

We later introduce a Memetic Algorithm (MA) for the node location problem in the localization field for the first time in the authors’ best knowledge with the characterization of time and noise uncertainties of LOS and NLOS conditions. There exist some previous studies of the application of the MA to the optimization of Wireless Sensor Networks (WSN) [43,44,45,46], but these studies are focused on the coverage among the system nodes.

In this paper, we are not only considering the effective coverage among the sensors, but also enhancing the performance of the LPS through the characterization of the system time and noise errors for designing competitive LPS for applications that demand high accuracy.

In this sense, the MA allows us to introduce knowledge in the optimization process through the concept of meme. A meme is the unit of cultural information of the Dawkins theory of transmittable knowledge [47] which has the ability to replicate and evolve and the capacity to affect the human fitness (i.e., reproduction and survival). This idea later inspired Moscato [48] to generate an impact in the evolutionary computation. The MA combines a local search strategy with the GA evolution for avoiding premature convergence. In addition, local search techniques are built to introduce knowledge in the optimization process for finding promising individuals in a reduced space of solutions, which may be difficult to be found in the GA evolution. However, a beneficial balancing among Global Search (i.e., GA performance) and Local Search (LS) is critical for achieving acceptable results in time and optimization [49].

We apply these ideas through a MA to the NLP with a variable neighborhood-descent LS in which the movement of the nodes for finding optimal sensor configurations is considered. The LS is applied to the most different individuals of the quantiles in which we divide the GA individuals through the fitness function evaluation. This allows us to explore new spaces of solutions not favored by the evolutionary process [50].

The variable neighborhood-descent LS implements a new pseudo-fitness function for characterizing promising node distributions in the reduced space of solutions of the LS in order to diminish the time complexity of the search. This is possible since geometric and clock errors are minority affected among contiguous solutions, and the notable increase of the fitness functions is produced by reducing NLOS links among the system elements.

Finally, we combine the beneficial effects of an enhanced GA operators selection during the evolutionary process in the HGA with the introduction of knowledge through the MA, obtaining a Hybrid Memetic Algorithm for the Node Location Problem which outperforms all the previous configurations. The process followed in the manuscript is detailed in the Graphical Abstract.

As a consequence, the main contributions of this paper are:

- A new ad-hoc hybrid combination of the GA operators in the evolutionary process for allowing a deep-exploration phase followed by a heavy-intensification phase for improving the results of the NLP.
- A MA methodology for the NLP in the localization field for the first time in the authors’ best knowledge.
- The construction of a pseudo-fitness function for improving the speed of the LS evaluation based exclusively on the positioning signal paths for evaluating neighborhood solutions of the NLP since geometric and clock errors remain practically constant among contiguous solutions.
- A variable neighborhood-descent LS not only for improving the best individuals of the GA population, but also for exploring the neighborhood of the most different individuals in order to analyze potential unfavored regions of the space of solutions due to the discontinuity of the fitness function in NLOS complex environments.

The remainder of the paper is organized as follows: we define the category and complexity of the NLP, the definition of the scenario of simulations and the CRLB model for the fitness evaluation in Section 2, the GA solution, its implementation and its weaknesses in the achievement of practical results in NLOS scenarios in Section 3, the HGA for introducing the ad-hoc usage of the GA operators for diversification and intensification phases in Section 4, the MA for the node location problem is introduced in Section 5, the results are shown in Section 6 while the conclusions of the paper are presented in Section 7.

## 2. Localization Node Location Problem

Let $\langle x_{i}\rangle =\left(x_{i},y_{i},z_{i}\right)$ be the spatial coordinates of a sensor node used for the localization in LPS, *S* the set of possible sensor locations in the environment (NLE region), $S_{j}$ a subset containing a possible combination of the defined *N* sensors used in the LPS located in different positions, $S_{l}$ the rest of the subsets of *S* excluding $S_{j}$, *T* the total possible target locations $\left(t_{k}\right)$ covered (TLE region), $f_{S_{j}}\left(t_{k}\right)$ the value of the fitness function of the optimization for the subset $S_{j}$ of sensors in a defined target location $\left(t_{k}\right)$, the node location problem is defined as finding the:

(1) $$\left(\langle x_{i}\rangle \;\left(i\in 1,\cdots ,N\right)=S_{j}\right)\subset S:\frac{\sum_{k=1}^{T}f_{S_{j}}\left(t_{k}\right)}{T}\geq \max\left(\frac{\sum_{k=1}^{T}f_{S_{l}}\left(t_{k}\right)}{T}\right)$$

Therefore, the Node Location Problem in localization entails the definition of the three Cartesian coordinates of the sensors used for localizing a TS in such a way that the fitness function of the quality of the system performance is maximized. This implies the combination of sensors that enables the reduction of the system uncertainties in the TS calculation for every analyzed point of the TLE in the optimization discretization. In this section, we address the category and complexity of the NLP, the definition of the scenario of simulation in which the GA, HGA and MA are applied and the model for the determination of the quality of a particular node distribution.

### 2.1. Category and Complexity of the NLP

The NLP has been categorized as NP-Hard [32,33,51,52], which shows the impossibility of finding the optimal solution of the problem in polynomial time without considering simplifications in the definition.

First attempts to address this problem were based on linear models applied on grid divisions of the NLE [53], which turned out to be very complex and required problem simplifications. As a consequence, non-linear models were proposed for finding valuable solutions without previous considerations through greedy algorithms [54].

However, the dimensions of the space of solutions did not allow us to solve the NLP with these methodologies achieving valid results, especially in discontinuous optimization spaces (e.g., NLOS system links considerations). Therefore, a heuristic solution to the NLP is recommended.

The main reasons are the non-derivability of the quality indicators for the complete TLE [27,31], the discontinuity of the space of solutions, the dimensions of the problem which depend on the resolution of the NLE and TLE regions and the complexity of the fitness function evaluation.

Simulated annealing [34], dolphin swarm [35], bat algorithm [36], elephant herding optimization [37], diversified local search [38], firefly algorithm [55], bacterial foraging algorithm [56] but especially genetic algorithms [39,40,41,57,58] have been used for solving the NLP.

GA have shown an excellent trade-off among diversification and intensification for this problem. Thus, they suppose the most extended methodology for the NLP in the literature, but we have found some problems for the exclusive evolutionary computation of the NLP that we will discuss in Section 3 and that recommend the introduction of some knowledge in the optimization process through a HGA and a MA.

However, regardless of the methodology used for the optimization, the complexity of the NLP must be considered for making beneficial design decisions. We define computational complexity of an algorithm as the amount of resources used for finding the optimal solution of a problem [59]. Considering the impossibility of solving the unconstrained problem (i.e., considering every possible sensor location), the complexity of the NLP depends on the characteristics of the resolution of the NLE [30]. The number of possible sensor distributions is defined as follows:

(2) $$P\left(\mathrm{Sensor}\;\mathrm{Distributions}\right)=\left[\prod_{i=0}^{N-1}\left(n_{NLE}-i\right)\right]$$

where $n_{NLE}$ is the total number of discretized points of the Node Location Environment which can hold an architecture sensor and *N* the total number of architecture sensors used in the LPS architecture.

Therefore, an increase in the number of architecture sensors used and a reduction in the resolution of the NLE induces the growth of the space of solutions. The order of the problem, as defined in Equation (2), is factorial.

In addition, localization NLP supposes the consideration of the analysis of the quality of a node distribution in every point of the TLE, since the optimal performance of the LPS must produce competitive results in the entire target coverage area. As a consequence, the total number of operations for considering the exhaustive analysis of every possible combination of sensors is:

(3) $$\mathrm{Number}\;\mathrm{of}\;\mathrm{operations}=\left[\prod_{i=0}^{N-1}\left(n_{NLE}-i\right)\right]n_{TLE}\;ff\left(t_{k}\right)$$

where $n_{TLE}$ is the total number of possible target locations analyzed for every possible sensor distribution and $ff(t_{k})$ is the function of the quality of a node distribution in a defined target location $\left(t_{k}\right)$.

Therefore, the time required for finding an optimized node distribution increases with the number of TLE analyzed points and it is dependent on the fitness function defined for the sensor distribution quality. This function will contain in this paper the combined uncertainties of noise in LOS and NLOS environments [21] and clock errors [20]. These effects are introduced in the covariance matrix of the FIM of the Asynchronous Time Difference of Arrival (A-TDOA) architecture.

The FIM matrix, for which the inverse is the CRLB of the system, is a maximum likelihood estimator in which the effect of optimal geometric deployments for the intersection of hyperboloid surfaces in TDOA localization is also considered. In this paper, we are also considering a pseudo-fitness function in the LS of the memetic algorithm since a reduction in the time complexity is achieved through the exclusive consideration of the LOS/NLOS links of the positioning signal paths.

The definition of these hyperparameters (NLE and TLE regions) for solving efficiently the NLP is discussed in the next subsection.

### 2.2. Definition of the Scenario of Simulations

**Figure 1:**
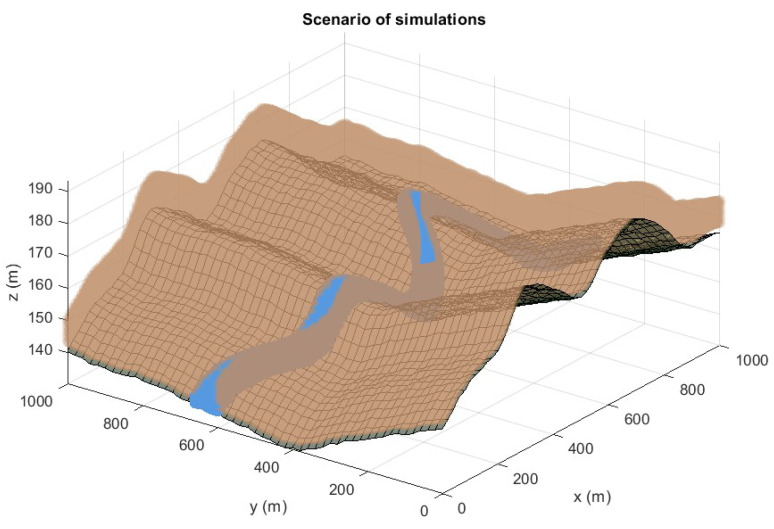
The scenario of simulations. Grey colors indicate the reference surface, blue colors represent the Target Location Environment (TLE) region, and brown zones show the Node Location Environment (NLE) region.

The proposed optimization technique for complex NLOS environments provides a potential way for a priori estimating the capabilities of positioning architectures deployed regardless of the conditions and the scenarios of application of the location systems. Under this assumption, this new optimization methodology should be tested in pre-defined 3D complex scenarios where NLOS discontinuities are induced, searching all the weaknesses and finding those variables that limit the future implementation of the procedure in other environments. In this aspect, a 3D scenario with harsh operating conditions and a base surface with obstacles and elevate ground slopes is presented in Figure 1.

Figure 1 shows the NLE and TLE regions of the designed scenario for all the simulations performed in the manuscript. This environment is unrealistic in terms of operating conditions and orography of the reference surface for the optimization, becoming a rough benchmark, and challenging the obtainment of adequate solutions for the deployment of sensors.

The TLE region extends in height from 0.5 to 3 m with respect to the base surface. TLE area is discretized under a spatial resolution of 10, 5, 1.5 m in the Cartesian coordinates *x*, *y*, and *z* respectively. With this configuration, the number of operations and thus the complexity of the problem is contained, maintaining higher consistencies and representativeness of the scenario. This is accomplished when the principal statistical variables of the accuracy evaluation of the positioning systems are slightly modified when increasing the spatial resolution of the TLE and NLE regions.

For the NLE zone, the architecture sensors are allowed in elevations from the local-based surface from 3 to 10 meters, in an attempt to maximize the conditions of adequate application of the CRLB model (avoiding multipath and other disruptive phenomena induced near the reference surface). The resolution in the NLE area is directly dependent on the codification of the sensor distributions in the individuals of the GA technique [30]. In this instance, a representation with binary chains of length 10,10,6 chromosomes for the respecting *x*, *y*, and *z*. Cartesian coordinates are selected, leading to resolutions of approximately 2 meters. As in the TLE region, this ensures a trade-off between representativeness of the results and the number of operations of the procedure.

### 2.3. Evaluation of the Quality of a Node Distribution

Localization NLP assumes an optimal sensor distribution for reducing the uncertainties in the determination of the TS location. The main system uncertainties in TBP are the noise degradation of the positioning signal in LOS and NLOS environments [21], the clock errors in the time measurements which are generated by synchronization of the system devices, drift and truncation errors in the CS clocks [20] and the geometric deployment of the sensors in space which affects the positioning algorithm performance [60].

The signal paths followed by the positioning signal vary notably in LPS. This fact recommends the usage of distance-dependent path-loss models for characterizing the signal path noises and for achieving practical results [29,31]. These models can be introduced in the covariance matrix of the FIM for characterizing the architecture errors. In addition, we proposed [20] a clock error model for considering the time uncertainties in the FIM covariance matrix which is used in this paper for achieving practical optimization results.

The definition of the FIM for for a time localization architecture was first proposed by Kaune et al. [25]:

(4) $$FIM_{mn}={\left(\frac{\partial h(TS)}{\partial TS_{m}}\right)}^{T}R^{-1}\left(\mathrm{TS}\right)\left(\frac{\partial h(TS)}{\partial TS_{n}}\right)+\frac{1}{2}tr\left\{R^{-1}\left(\mathrm{TS}\right)\left(\frac{\partial R(TS)}{\partial TS_{m}}\right)R^{-1}\left(\mathrm{TS}\right)\left(\frac{\partial R(TS)}{\partial TS_{n}}\right)\right\}$$

where ***R(TS)*** is the covariance matrix of the architecture at study in which the characterization of the uncertainties (i.e., noise in LOS/NLOS condition and clock errors) is provided and ***h(TS)*** is the vector containing the information of the time measurement computed in the A-TDOA architecture.

Particularizing ***h(TS)*** and ***R(TS)*** for the A-TDOA architecture [20,21] and assuming uncorrelated time measurements in the A-TDOA architecture [7]:

(5) $$h_{A-TDOA_{i}}=∥TS-WS_{i}∥+∥TS-CS∥-∥WS_{i}-CS∥\;i=1,\cdots ,N_{WS}$$

(6) $$\begin{array}{c} \sigma_{A-TDOA_{i}}^{2}=\frac{c^{2}}{B^{2}\left(\frac{P_{T}}{P_{n}}\right)}PL\left(d_{0}\right)\left[\left(\frac{d_{WS_{i}-TS_{LOS}}}{d_{0}}\right)+{\left(\frac{d_{WS_{i}-TS_{NLOS}}}{d_{0}}\right)}^{\frac{n_{NLOS}}{n_{LOS}}}+\left(\frac{d_{TS-CS_{LOS}}}{d_{0}}\right)+\right.\\ {\left.+{\left(\frac{d_{TS-CS_{NLOS}}}{d_{0}}\right)}^{\frac{n_{NLOS}}{n_{LOS}}}+\left(\frac{d_{WS_{i}-CS_{LOS}}}{d_{0}}\right)+{\left(\frac{d_{WS_{i}-CS_{NLOS}}}{d_{0}}\right)}^{\frac{n_{NLOS}}{n_{LOS}}}\right]}^{n_{LOS}}+\\ +\frac{1}{l}\sum_{k=1}^{l}\left\{\left|\left(T_{i}+T_{TS}-T_{CS}\right)-floor_{TR}\left[\left(T_{i}+T_{TS}-T_{CS}\right)\left(1+\eta_{CS}\right)\right]\right|c^{2}\right\} \end{array}$$

(7) $$d_{WS_{i}-TS_{LOS}}={∥WS_{i}-TS∥}_{LOS}$$

(8) $$d_{WS_{i}-TS_{NLOS}}={∥WS_{i}-TS∥}_{NLOS}$$

(9) $$d_{TS-CS_{LOS}}={∥TS-CS∥}_{LOS}$$

(10) $$d_{TS-CS_{NLOS}}={∥TS-CS∥}_{NLOS}$$

(11) $$d_{WS_{i}-CS_{LOS}}={∥WS_{i}-CS∥}_{LOS}$$

(12) $$d_{WS_{i}-CS_{NLOS}}={∥WS_{i}-CS∥}_{NLOS}$$

where sub-index *i* represent the measurements and signal paths linked with architecture sensor *i*, while $N_{WS}$ represents the number of Worker Sensors (WS); *c* is the speed of the radioelectric waves in m/s, *B* the signal bandwith in Hz, $P_{T}$ the transmission power in W, $P_{n}$ the mean noise level in W calculated through the Johnson–Nyquist relation, $PL(d_{0})$ the path-loss in the reference distance $d_{0}$ from which the Log-Normal model is considered; $d_{WS_{i}-TS}$, $d_{TS-CS}$, $d_{WS_{i}-CS}$ the LOS and NLOS distances travelled from the WS to the TS, from the TS to the CS and from the WS to the CS respectively calculated with the algorithm described in [21]; $n_{LOS}$ and $n_{NLOS}$ the path-loss exponents used in the Log-Normal model, *l* is the number of iterations of the Monte–Carlo simulation performed for estimating the temporal variances, $T_{i}$, $T_{TS}$ and $T_{CS}$ the time of flight of the positioning signal from the TS to the system WSs, the duration of the flight from TS to the CS and the period of time from the emission of the signal from the WS to the TS respectively; $\eta_{CS}$ define the clock drift of the CS clock.

This FIM characterization allows us to consider the main architecture uncertainties in the optimization process of the NLP and finally obtain a measurement of the minimum achievable error achieved by any positioning algorithm through the trace of the inverse of the FIM, expressed through the Root Mean Squared Error (*RMSE*) as the most spreaded accuracy metric:

(13) $$RMSE=\sqrt{\sum_{m=1}^{n}FIM_{mm}^{-1}}$$

## 3. Genetic Algorithm for the NLP in Localization

Genetic Algorithms have shown an excellent trade-off between diversification and intensification for the NLP. These GA were proposed by Holland [61] and later refined by Goldberg [62]. They are built on the theory of evolution and rely on the characteristics of the descendants of a population which present a better adaptation than their parents by receiving the adapted genes from the previous generation. The usage of the GA operators allows the recombination of the individuals, the selection of the best candidates for finding an optimal offspring, the mutation of some genes for exploring new spaces of solutions avoiding local optima and the elitism for preserving the best adapted individuals from generation to generation.

**Figure 2:**
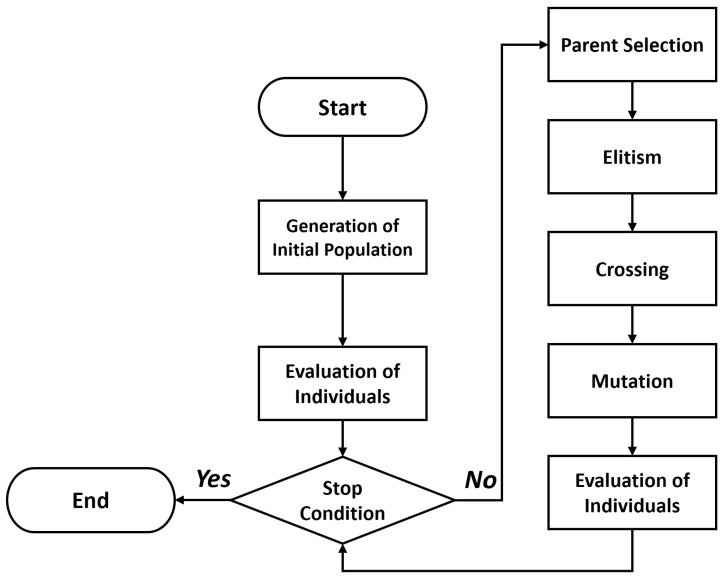
Flux Diagram of a Genetic Algorithm (GA).

**Figure 3:**
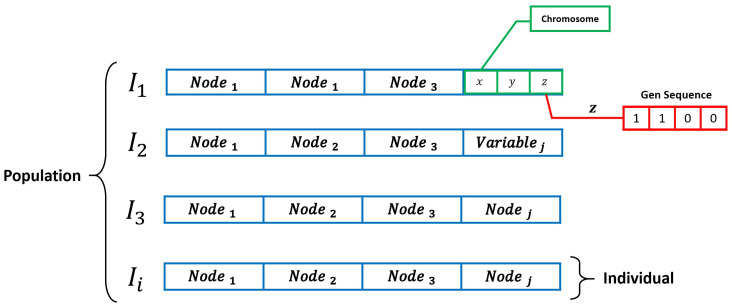
Binary codification of the GA for the Node Location Problem (NLP).

We provide in Figure 2 and Figure 3 a general framework of the GA performance and based on the binary codification proposed in the original work of Holland [61] (i.e., a candidate node distribution for the NLP).

As it is shown in Figure 3, the variables to optimize in the NLP are the Cartesian coordinates of each architecture sensor node (i.e., the chromosomes of the codification) and the definition of the resolution of the optimization allows us to transfer the binary coding of the potential solution to decimal numbers through the escalation process defined in [30]. The definition of the quality of every individual of the GA is based on a fitness function considering the CRLB described in Section 2.3 which enables the application of pressure selection for allowing the evolutionary process find an optimal sensor configuration. The achievement of valid solutions in the GA performance requires an exhaustive definition of the hyperparameters of the optimization [63]. In this section, we analyze the potential problems of the NLP optimization through GA and propose two potential solutions through HGA and MA.

### 3.1. Implementation of the GA

Therefore, we have implemented a GA configuration that aims to find the best possible distribution of sensors. For the scenario proposed, shown in Figure 1, in Table 1 a set of generic technology parameters have been selected for performing simulations. The reason for this selection relies on the main objective of this research, which is the generation of a new optimization technique to the NLP, not the resolution of the NLP for a particular positioning technology.

Once the initial conditions of the optimization are established, the number of sensors to achieve the desirable accuracy of the LPS is studied. This is a critical aspect in the NLP since an insufficient number of sensors may lead to coverage issues and unacceptable RMSE values in some TLE analyzed points. On the other hand, an unnecessary amount of nodes shall incur in a considerable increase on the system implementation and maintenance cost.

Therefore, we have designed a genetic algorithm that obtains through the evaluation of a fitness function the optimal node distribution and performance for multiple numbers of sensors. The genetic algorithm, whose hyperparameters are shown in Table 2, is instructed by the following fitness function.

(14) $$ff=1-{\left(\frac{\bar{RMSE}}{RMSE_{ref}}\right)}^{2}$$

where $\bar{RMSE}$ is the mean value of the *RMSE* of a certain individual or node distribution for every possible target location (i.e., each of the TLE analyzed points). On the other hand, $RMSE_{ref}$ is a defined hyperparameter of the GA and serves as an accuracy reference [7,21]. This control parameter represents the maximum RMSE value that can be reached for the TLE by an individual node distribution. Reducing the $RMSE_{ref}$ shall introduce pressure selection in the optimization process, improving the overall result. However, a disproportionate value may compromise the convergence of the GA to any solution, therefore it is critical to obtain an adequate value for each particular scenario.

Furthermore, due to the construction of the fitness function in Equation (14), all fitness values should be represented in the interval [0,1]. Therefore, the value selected for the $RMSE_{ref}$ must ensure that every fitness evaluation remains in the desired region.

Hence, in Table 3 we study the performance of the GA under different node distributions in search of the most adequate configuration respecting performance and costs of the system. From these results, it is concluded that the best compromise solution regarding the systems performance and costs is an 11-node distribution. A lower number of sensors shall incur in a greater and unfeasible positioning errors. On the other hand, a higher number of nodes does not accomplish a significant improvement in the positioning accuracy, thus proving the investment on additional sensors futile.

### 3.2. Weaknesses of the GA Optimization in the NLP

GA evolution is a heuristic process in which randomness allows us to explore potential regions of the space of solutions for finding an optimized solution, but the results achieved may vary among different runs since the introduction of the same inputs do not produce the same results. This is a consequence of the evolutionary process in which two phases can be defined: diversification and intensification.

In the first stage, the GA looks for promising regions in which an optimal solution can be found (i.e., diversification). Later, an exhaustive search in the promising regions (i.e., intensification) is promoted for finding the best adapted individual of these regions.

The mutation of some individuals is required for exploring new regions and avoiding local optima. However, the new individuals produced in the mutation operation must be good enough to hold the pressure selection. Otherwise, these individuals will disappear even if they belong to really promising regions. Even, the finding of new promising regions can be affected in especially discontinuous fitness function regions since the evolutionary process may suffer problems to reach the local optima if a deep increase in the fitness function can be produced among contiguous solutions (e.g., NLOS environments by the avoidance of obstacles in the positioning signal links). Therefore, we can affirm that the mutation process depends also on randomness and the exploration of new potential regions in discontinuous optimizations may be limited by the evolutionary pressure selection.

Thus, GA optimization in especially huge spaces of solutions such as in NLP optimizations in which NLOS links are considered may suppose a challenge in which the results can notably vary among different runs and the exploration of the space of solutions supposes an actual threat. As a consequence, we propose the introduction of knowledge in the optimization for solving these potential weaknesses in the GA optimization in the NLP.

Firstly, we introduce a HGA for taking advantage of the usage of the GA operators in Section 4. We generate diversity in the generation of the new individuals in the diversification and intensification phases for achieving better optimization results. We later propose a MA with a variable neighborhood-descent LS in which we introduce a methodology for detecting the most different individuals (i.e., new potential spaces of solutions) and we analyze its local region of potential solutions for allowing the finding of promising solution in discontinuous spaces in Section 5.

## 4. Implementation of Hybrid Genetic Algorithm in the NLP

The performance of every GA optimization is heavily dependent on the balance between the diversification and intensification capabilities of the GA. These values are established by the genetic operators utilized in the GA configuration, such as the selection and crossover operators, along the hyperparameters selected. An adequate equilibrium between these two competences is essential in favor of obtaining the optimal solution to the NLP.

An excessive focus on the intensification aspect, despite facilitating the convergence to the solution, may diminish the results obtained since relatively none exploration of the solution environment has been made. On the other hand, a disproportionate commitment on the diversification capability shall boost the entropy of the optimization to a point where the convergence to a solution is compromised or even unfeasible.

Therefore, the balance between these two capabilities is crucial for the optimization performance, hence the configuration of genetic operators must be selected accordingly.

HGA have received a growing interest throughout the GA literature, being utilized for solving real-world problems [36]. HGA open up new possibilities as they support multiple configurations of genetic operators and hyperparameters.

Thus, HGA are idoneous for applications where the solution environment is notably unfavorable. Scenarios that contain a consequential number of local maximums, such as the one studied in this paper, require both diversification, towards locating the global maximum region, and intensification, in order to obtain the optimal value of that region.

Therefore, for these particular scenarios, the approach of utilizing a HGA composed of multiple phases of diversification and intensification of the solution may exceed any achievable solution obtainable by any individual combinations of genetic operators.

Accordingly, in Table 4 we analyze the performance of multiple combinations of genetic operators in search of the most appropriate configuration for this particular scenario.

Results in Table 4 show that the most appropriate techniques are the combination of tournament 2 (T2) selection criteria and multi-point crossover with 3 crossover points (MP3), along the roulette (R) selection methodology with also the MP3, exceeding these two combinations any other configuration.

T2 and especially Roulette are particularly elitist techniques [66], hence we can conclude that for the scenario proposed, a heavy approach on intensification is far more advantageous than a diversification-focused methodology.

However, the T2–MP3 combination achieves a greater exploration of the solution environment. Thus, it is possible to elaborate a HGA that utilizes both methodologies in search of a greater solution.

**Figure 4:**
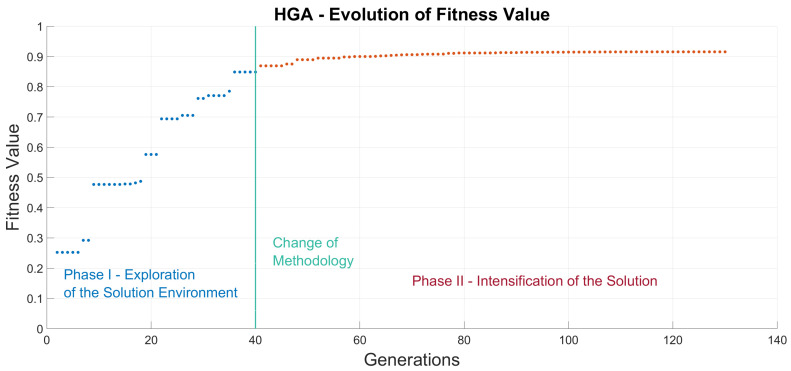
Hybrid Genetic Algorithm (HGA) optimization for the node location problem.

Consequently, in this paper we propose the configuration of a HGA that relies in two different phases, a deep-exploration phase followed by a heavy-intensification phase. The first phase incorporates a tournament 2 selection criteria along a three-point crossover and aims to explore the depth of the solution environment in search of the global maximum. Afterward, a second combination of roulette selection methodology and also three-point crossover seeks to obtain an improved solution with regards to GA optimizations, as shown in Figure 4.

Results in Table 5 prove that indeed a HGA approach that combines two different phases may exceed the results obtained by any individual combination of genetic operators.

Although it is true that HGA can exceed GA configurations, especially in adverse scenarios, the implementation of a HGA require the adjustment of a considerable amount of hyperparameters in addition to a profound analysis on the methodologies and genetic operators selected, which can only be done experimentally.

Therefore, it is critical to analyze each particular situation, as to determine if the implementation of an ad-hoc HGA configuration is in order.

In conclusion, HGA are a promising alternative to GA, and may surpass the results traditionally obtained by these algorithms, especially for adverse scenarios. However, the performance of the HGA is susceptible to the adequate selection of the genetic operators and the values of the hyperparameters inherent to this algorithm, depending this selection on each particular situation (i.e., the scenery of simulations).

However, it is possible to elaborate a different strain of heuristic algorithm that provides both intensification and diversification capabilities along a solid versatility between different scenarios. In the next section, we will study and analyze the implementation of a MA to the NLP.

## 5. Implementation of Memetic Algorithms and Local Search to the NLP

Within the compendium of metaheuristic methodologies, Memetic Algorithms are characterized by their inclusion of the problem’s knowledge into the solution optimization. Consequently, the incorporation of particular information of the problem may achieve greater results than the previous methodologies introduced.

Moreover, once the optimization process for the NLP is studied and particularized, the resulting MA achieves a higher versatility than GA or HGA. Even though it is possible to modify the initial conditions or the current scenario of study, all these applications share the foundations of the NLP whose knowledge is integrated into the MA optimization. The foundations of the MA are discussed in the next subsection and the implementation to the NLP subsequently.

### 5.1. Fundamentals of Memetic Algorithms

In this paper we have introduced the complexity of the NLP, consequently, a GA optimization was proposed in virtue of its diversification capabilities, which result vital in the optimization process, especially for adverse scenarios (e.g., the one studied in this paper).

Nonetheless, it is possible to implement a different heuristic methodology that allows a higher versatility along achieving possibly greater results, such is the case of Memetic Algorithms (MA), which we will analyze forthwith.

MA combine the optimization process of a GA along a LS technique. Through the LS methodology, we introduce the knowledge of the problem, in search of the most promising individuals within a reduced solution environment, which may pass unsighted in the GA evolution.

Although there exists some former studies of MA optimization for Wireless Sensor Networks [43,44,45,46], these studies take only into account the coverage among the sensors. In this paper, we will implement a MA for the NLP for the first time in the author’s best knowledge with time and noise uncertainties characterization in the localization field.

### 5.2. Memetic Algorithm Structure

The Memetic Algorithm combines both Global Search (i.e., GA optimization) and Local Search in pursuit of exceeding the results obtained by any of these methodologies individually. Therefore, we propose the following codification of a MA for the NLP.

**Figure 5:**
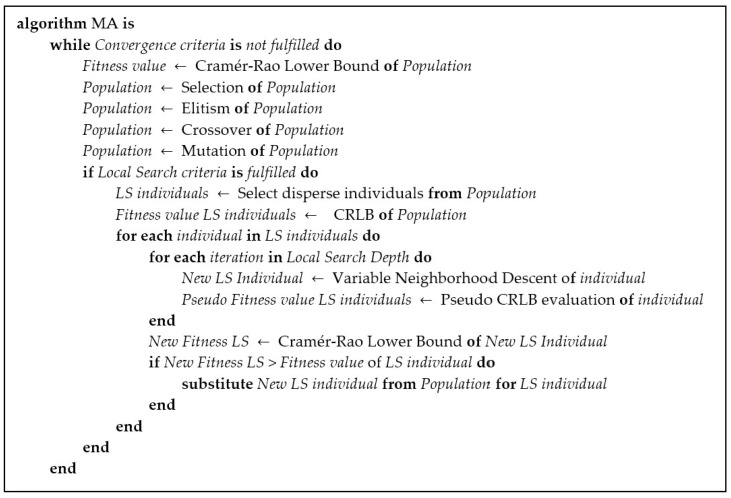
Memetic Algorithm (MA) Pseudo-code.

Figure 5 shows the structure of the MA implemented for the NLP. The MA is composed of a GA optimization and the corresponding genetic operators along the LS methodology. For the NLP, we propose a variable neighborhood-descent LS technique where the position of the nodes above the terrain is considered, thus introducing some knowledge of the problem into the optimization process.

Once the population is mutated, the algorithm determines whether to proceed with the LS methodology for each generation. This depends on the LS frequency [49] which must be balanced in the combination of Global and Local Search for achieving the optimal optimization results in MA. It is vital to execute the LS after the mutation have finished, on the contrary, the progress made in the LS may be lost by the mutation of the population. Both the possibility of executing a LS and the number of individuals examined are hyperparameters that must be studied.

If the MA proceed with the LS technique, the first step through this algorithm is the selection of the most diverse individuals. The LS methodology pretends to explore and intensify undiscovered regions by the GA where a local or global maximum may be located, therefore it is vital to select a certain number of individuals that are distant within each other in order to explore the maximum space of solutions possible.

Hence, we have developed a branch and bound algorithm that analyzes the population in search of the individual whose dissimilarity within each other are the greatest, thus optimizing the results obtained, which we will discuss in Section 5.3. Once the dissimilarity of each individual is evaluated, the most diverse are transferred into the variable neighborhood-descent LS technique.

The variable neighborhood-descent LS evaluates reduced movements of the node positions for each individual (i.e., contiguous solutions in the neighborhood of the individual) in a new pseudo-fitness function in order to reduce the time complexity of the analysis. This procedure does not compromise the optimization since reduced movements of the sensors shall not incur in considerable deviation of geometric or clock errors. On the contrary, the pseudo-fitness function proposed is adequate for detecting NLOS trajectories that diminish the fitness value of the localization architecture. Therefore, this LS methodology excels in particularly adverse scenarios, where NLOS trajectories are considerable (e.g the one studied in this paper). In these scenarios, minimal changes in the node locations may result in considerable deviations of the fitness function since the avoidance of an obstacle may suppose a significantly increase in the localization accuracy.

Consequently, if in a certain direction an increase in the fitness function is detected, the new improved individual shall substitute its predecessor. Hence, the LS technique proposed can only improve the fitness function of the individuals analyzed, thus improving the overall performance of the optimization.

The LS technique in the MA introduces a spike of diversity and intensification into the optimization process. This effect shall prove useful when the GA convergence is compromised as a fact of the existence of local maximums, resulting in an overall greater performance of the optimization, achieving consequently better solutions.

However, within the LS technique there exists an abundant quantity of algorithms on which the development and configuration of the performance of the MA relies. Therefore, we shall analyze it thoughtfully forthwith.

### 5.3. Local Search in the MA Optimization

The LS method grants to obtain accurate information about a bounded region defined by a distance function on the space of solutions. LS explores near neighbors for finding the best-adapted individuals within the area. Every set of adjacent individuals or distance 1 defines the neighborhood. Once the aim number of neighbors has been inspected, the next point in the LS is the selection of the best fitness neighbor. The algorithm ends when the stopping condition is reached (e.g., there is no evolution in the fitness between generations or the neighbor reached satisfies a criterion) or when over the maximum number of local iterations permitted is attained.

During the execution of the LS, the optimization of the number of individuals, breadth of search, and the count of depth iterations are vital factors for achieving practical results. Different LS techniques are considered in the literature, such as Tabu Search [67], Variable Neighborhood-Descent (VND) [68], selective LS [69], LS chain [70], or Iterated LS [71]. The adaptation to the characteristics of the problem determines their selection.

In this paper, VND is chosen since it allows the quantification of the improvement of the fitness in the spatial directions of the sensors in their neighborhood (i.e., the proximal allowable locations of the architecture sensors) for defining a path in the LS optimization. The application of LS in MA is critical for introducing knowledge in the evolutionary optimization process. Previous researches have used LS for introducing heterogeneity in the final solution for improving the elite individuals of the population [68] or for accelerating the overall speed of the optimization.

In this paper, we use LS in the MA not only for improving the elite individuals, but also for introducing diversity in the evolutionary process for examining potential unfavored spaces of solutions. Potential unfavored areas of solutions appeared in the NLP in NLOS conditions. Significant differences in the fitness values are produced among contiguous solutions since obstacles significantly modify the architecture noises of adjacent node distributions.

The LS enables the examination of the most different individuals of the population to find potential optimum node distributions that are difficult to access through the GA operators and the evolutionary process.

#### 5.3.1. Pseudo-Fitness Function

A critical issue in the MA is the selection of a LS fitness function, which should be kept in harmony with the GA search function [72]. The GA presented in Section 3 proposes the minimization of the CRLB error characterization of the TDOA architecture. However, we propose a pseudo-fitness function in the LS which analyzes the LOS/NLOS links of the positioning signal paths.

The pseudo-function allows the finding of the optimum node distribution of reduced search spaces defined by neighborhood relations. Pseudo-function is composed by a path loss exponent value of the LOS and NLOS links and the total distances of the LOS and NLOS links under coverage which are used for the target location determination. The reduction of the paths allows the minimization of the noise uncertainties which supposes the main error source among neighboring potential solutions.

(15) $$ff_{LS}=\frac{1}{\sum_{k=1}^{T}\sum_{i=1}^{N}\left[d_{i_{LOS}}^{n_{LOS}}+d_{i_{NLOS}}^{n_{NLOS}}\right]}$$

This optimization methodology has led to the maximization of the inverse of the sum values associated with the LOS and NLOS links in each possible TLE analyzed point *T* for each architecture sensor under coverage *N*.

This pseudo-function has proven its competence to ensure the finding of the neighborhood local optima. A new neighborhood for the next LS iteration is defined through the selection of the most adapted individual of the neighbors analyzed based on the pseudo-fitness function values. The definition of a different fitness evaluation for the LS instead of the fitness used in the GA optimization promotes the analysis of the CRLB of the local optima individual of the neighborhood just before introducing the LS optimal to the general optimization process.

#### 5.3.2. Variable Neighborhood-Descent Local Search

The neighborhood search aims to maximize the pseudo-fitness function to obtain the local optima of the LS individual selected. Since the geometric and clock errors remain practically constant among contiguous solutions, the neighborhood LS looks for reducing the NLOS paths of the positioning signals.

In this paper, we apply a variable neighborhood-descent algorithm (VND) [73] which finds the best individual of a defined neighborhood and later defines a new neighborhood based on the current LS individual optimum. VND is constantly improving or keeping the best LS individual in a new neighborhood for a maximum defined number of iterations, which is known in MA as Local Search Depth (LSD).

VND algorithm can also end by finding an individual sufficiently improved (e.g., avoiding all the NLOS connections in the positioning signals). The LS exploration is performed for each sensor of the architecture. The neighborhood is defined for every sensor which is moved around its neighborhood for improving its positioning connections. We explore 26 potential movements of each sensor for improving the pseudo-fitness function value (i.e., 26 directions are considered for every sensor in each iteration of the LS). This LS is particularly crucial for the CS since this sensor is used for computing the time measurements in the A-TDOA and consequently the positioning links of the CS affect the quality of a node distribution in a bigger extent.

#### 5.3.3. Definition of the LS Individuals

The application of the MA LS in this article looks for providing genetic variability in the population and for discovering unexplored regions. In the intensification phase of the GA optimization, the existence of many individuals in a defined domain of the space of solutions promotes the access to every possible NLE solution through the crossover operator in this area. Nevertheless, GA make the most different individuals in this final optimization stages very probable to disappear without the exploration of their surrounding region thoroughly.

In addition, the performance of the GA mutation in any optimization phase, which can produce diverse individuals, is not enough for providing variability in NLOS environments since the exploration of new potential regions is limited to the finding of a good enough individual in the new space of solutions to survive the pressure selection of the next generation. Therefore, we use the MA LS to explore the most different individuals of the population in order to find new promising solutions to the NLP.

**Figure 6:**
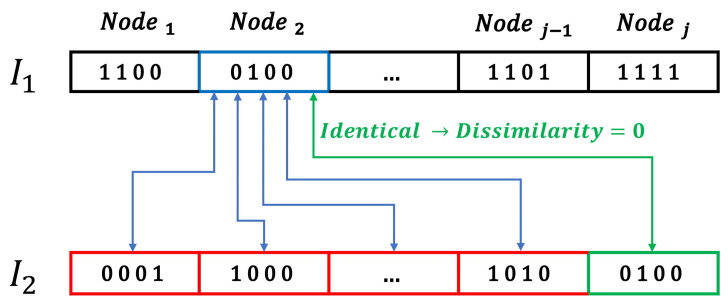
Calculation of the dissimilarities of Node 2 of Individual 1 $\left(I_{1\;N_{2}}\right)$ with each node of the Individual 2 $\left(I_{2}\right)$. The dissimilarity between $I_{1\;N_{2}}$ and $I_{2\;N_{j}}$ is zero since they are identical and is determined through the Hamming distance with the rest of the nodes of $I_{2}$.

The definition of the most different individuals of the population is achieved through the measurement of the dissimilarity among solutions. The dissimilarity is calculated by applying the Hamming distance in the binary codification of two different solutions (i.e., two different sensor distributions) [74]. However, the dissimilarity metric cannot be directly applied since identical sensors can be located in different positions of the binary codification of two different individuals. Hence, each sensor of any individual must be first compared with all the sensor locations of the rest of the individuals, as it can be seen in Figure 6.

Therefore, the measurement of the dissimilitude among the different sensor distributions requires the finding of the pairs of sensors among two different potential solutions ($I_{1}$ and $I_{2}$) which are more similar among them. However, greedy approaches cannot be applied for achieving this value since not the selection of the most similar nodes of two different individuals provides the minimum sum of the Hamming distance among the individuals. Consequently, we define the dissimilarity matrix among individuals *d* containing the values of the Hamming distance of each node of the $I_{1}$ with each node of the $I_{2}$:

(16) $$d=\left(\begin{array}{ccccc} d(I_{1\;N_{1}},I_{2\;N_{1}}) & d(I_{1\;N_{1}},I_{2\;N_{2}}) & \cdots & d(I_{1\;N_{1}},I_{2\;N_{j-1}}) & d(I_{1\;N_{1}},I_{2\;N_{j}})\\ d(I_{1\;N_{2}},I_{2\;N_{1}}) & d(I_{1\;N_{2}},I_{2\;N_{2}}) & \cdots & d(I_{1\;N_{2}},I_{2\;N_{j-1}}) & d(I_{1\;N_{2}},I_{2\;N_{j}})\\ \vdots & \vdots & \ddots & \vdots & \vdots \\ d(I_{1\;N_{j-1}},I_{2\;N_{1}}) & d(I_{1\;N_{j-1}},I_{2\;N_{2}}) & \cdots & d(I_{1\;N_{j-1}},I_{2\;N_{j-1}}) & d(I_{1\;N_{j-1}},I_{2\;N_{j}})\\ d(I_{1\;N_{j}},I_{2\;N_{1}}) & d(I_{1\;N_{j}},I_{2\;N_{2}}) & \cdots & d(I_{1\;N_{j}},I_{2\;N_{j-1}}) & d(I_{1\;N_{j}},I_{2\;N_{j}}) \end{array}\right)$$

We explore the *d* matrix through a branch and bound algorithm [75] for finding the combination of sensors of the two individuals which minimizes the Hamming distance of the pair of individuals. The procedure follows the definition of the most promising node (i.e., the more reduced value of the *d* matrix) and later exploring the possible combinations of the matrix without repeating row and column for finding the pairs of sensors which minimizes the sum of the dissimilarities. Once these pairs of similar sensors have been defined, the dissimilitude among solutions is defined as:

(17) $$D_{ij}=\min\left(\sum_{pair}^{N}d_{{hamming}_{pair}}\right)$$

where $D_{ij}$ is the dissimilitude among the solutions *i* and *j*, $d_{{hamming}_{pair}}$ is the hamming distance measured in one of the pairs of contiguous sensors among solutions previously defined and *N* the total number of sensors used for the localization.

Once the dissimilitude among solutions is defined, it can be expressed in matrix form *D* for a general definition of the distances among every of the population individuals:

(18) $$D=\left(\begin{array}{ccccc} 0 & D_{1,2} & \cdots & D_{1,(n-1)} & D_{1,n}\\ D_{2,1} & 0 & \cdots & D_{2,(n-1)} & D_{2,n}\\ \vdots & \vdots & \ddots & \vdots & \vdots \\ D_{(n-1),1} & D_{(n-1),2} & \cdots & 0 & D_{(n-1),n}\\ D_{n,1} & D_{n,2} & \cdots & D_{n,(n-1)} & 0 \end{array}\right)$$

The finding of the most different individual of the set to whom we apply the LS in search for new unexplored spaces of solutions is obtained through the maximization of the *D* matrix row or column sum values since the dissimilarity matrix is symmetric. This sum represents the total difference of an individual with the rest of the individuals of the population. According to this total dissimilitude factor, the population is ordered and a percentage of the first new individuals (i.e., the most different) is chosen for executing the VND algorithm.

In addition, we select the elite individual of the population for practicing the LS on it, thus obtaining an improvement in the accuracy results of the optimization within the LS.

## 6. Results

In this section, we present the results obtained by the MA optimization introduced in the previous section, along with some comparisons with previously proposed methodologies. All algorithms and simulations were coded and executed in the Matlab software environment, being every test performed with an Intel(R) i7 2.4 GHz CPU and 16 GB of RAM.

Table 6 shows the results of the MA optimization. Due to the overall performance improvement of the optimization achieved by the LS, the final node distribution obtained reach a significant increase in positioning accuracy from previous simulations from Table 3. Therefore, in pursuit of the optimal compromise between position accuracy and amount of sensors (i.e installation and maintenance costs) we can lower the number of sensors to 8 nodes without compromising the system accuracy.

**Figure 7:**
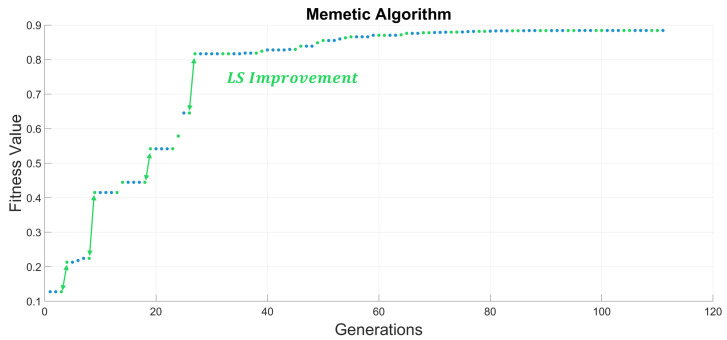
Memetic Algorithm convergence to the optimal solution for an 8-node distribution. Results show that the Variable Neighborhood-Descent (VND) utilized in the LS algorithm introduces significant improvements on the fitness values of the selected individuals, thus improving the convergence. These improvements escalate rapidly due to the effect of elitism on the enhanced individuals, thus preserving and spreading even further their properties.

Figure 7 shows the MA search of the optimal solution, combining the GA optimization with a LS methodology that enhances the overall performance of the optimization with every iteration of the VND. However, it is possible to improve even further the MA optimization, relying this technique on a GA, thus a single combination of genetic operators and hyperparameters. Furthermore, it is possible to implement multiple configurations of genetic operators and hyperparameters into the GA optimization, thus obtaining a HGA, that along the LS of the MA results in an overall improvement of the optimization performance carried out by the proposed Hybrid Memetic Algorithm (HMA). Therefore, Table 7 shows the positioning error for each methodology proposed. Hence, we can appreciate an escalated increase in the optimization results with each step forward in the methodology selected.

**Figure 8:**
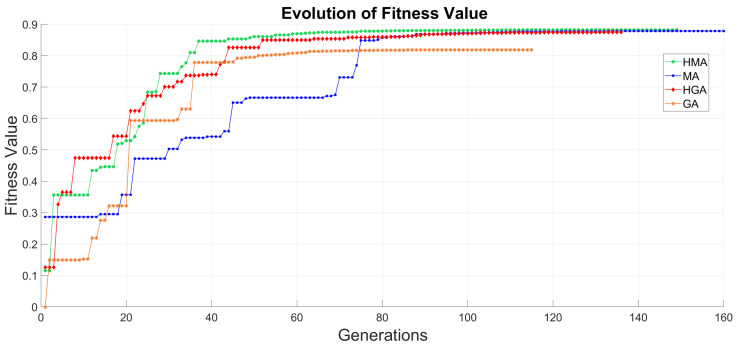
Convergence of the compendium of techniques proposed in this paper for 8 nodes.

Furthermore, Figure 8 shows the compendium of techniques introduced in this paper and their respective convergence for an 8 nodes distribution optimization. This Figure proves the importance of an adequate selection of genetic operators and optimization methodologies. The optimization of the fitness value requires both diversification, in order to obtain an overall greater solution, and intensification, thus enhancing the convergence.

The hybrid algorithms proposed show a steadier convergence, as well as an overall greater result, demonstrating a greater balance between intensification and diversification. Besides, the MA achieves overall greater results than previous methodologies, proving the superior optimization performance of the MA and HMA.

**Figure 9:**
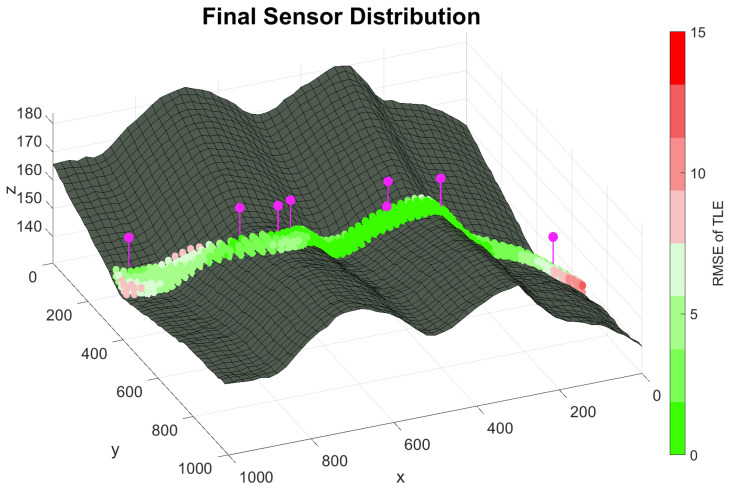
Optimal node distribution of 8 sensors obtained by the Hybrid Memetic Algorithm (HMA) and the RMSE accuracy of the TLE for the scenario proposed. The obtained distribution achieves an overall adequate positioning against the terrain adversities, even so for a reduced number of sensors.

Ultimately, Figure 9 show the optimal node distribution obtained by the HMA in Table 7, along the RMSE values for the TLE.

From the obtained results, we can conclude that the resulting increase in diversification introduced by the MA derives in an increase in the number of generations the final convergence to a solution. However, as shown in Figure 8 this additional diversity implemented into the optimization achieves higher results than other methodologies as it allows a greater exploration of the solution environment.

Nevertheless, the implementation of a MA requires the introduction of knowledge into the problem, thus investing additional time into designing a specific LS methodology for each different problem. The MA excels when faced against extremely adverse scenarios, or against different initial conditions that may turn ineffective the hyperparameters previously adjusted.

Therefore, it is critical to analyze each particular case, taking into consideration the complexity of the scenario along the possible variability of their initial configuration, in search of the optimal methodology for each particular case. Nevertheless, as the results show, MAs are ideal versatile techniques for adverse variable scenarios, such as the one proposed in this paper.

## 7. Conclusions

Local Positioning Systems (LPS) are attracting large research interest over the last few years for performing applications that demand high accuracy, such as guided autonomous navigation in indoor and outdoor environments. Availability, robustness, hardware configuration, architecture coverage and uncertainties reduction are some of the most important issues addressed for achieving optimal sensor node deployments and fulfilling the LPS design requirements.

These tasks require optimized ad-hoc node distributions for adapting to the characteristics of the environment in which the LPS are deployed. Among LPS, those based on temporal measurements stand out since they provide a relevant trade-off among costs, hardware complexity, robustness and accuracy. The achievement of valid node deployments in Time-Based Positioning Systems (TBS) demands an error characterization in which the noise of the communications channel in LOS and NLOS architecture links and the clock errors in the temporal measurements must be considered.

The TBS have shown an excellent performance for LPS applications and among these architectures novel asynchronous architectures stand out due to the unnecessary synchronism of the system devices consequently reducing the clock errors. Thus, in this paper, we define a Cramér–Rao Bound (CRB) model for the Asynchronous Time Difference of Arrival (A-TDOA) architecture since CRB provides the minimum achievable positioning error of this architecture by using any positioning algorithm.

This CRB model is applied for measuring the quality of an A-TDOA node deployment for solving the Node Location Problem (NLP) of this architecture. The NLP requires the finding of the optimized cartesian coordinates of the architecture sensors of any sensor network distribution. It has been assigned as NP-Hard since a polynomial or exact solution cannot be found. Therefore, a heuristic solution to the NLP has been extended in the literature. Amongst the metaheuristic techniques, Genetic Algorithms (GA) have shown an excellent trade-off between the diversification and intensification stages of the optimization.

However, in our previous research we have found that the GA optimization is unstable in NLOS environments in which the discontinuities in the fitness values of contiguous solutions makes the exploration of new potential spaces of solutions be difficult to address.

As a consequence, in this paper we propose the introduction of knowledge in the optimization process. First, we propose a hybrid GA (HGA) based on the modification of the GA operators during the optimization process defining two optimization phases: an enhanced deep-exploration phase followed by a heavy-intensification phase.

Later, we introduce for the first time in the authors’ best knowledge a memetic algorithm (MA) consisting of a mixture of the GA optimization with a variable neighborhood-descent (VND) Local Search (LS) strategy for the NLP in the localization field. The MA applies the LS to the most different individuals of the population defined by Hamming distance in order to explore new different spaces of solutions not favored by the evolutionary optimization process. In addition, we define a pseudo-fitness function based on the reduction of the architecture LOS and NLOS links since geometric and clock errors have a reduced impact in the neighborhood in which the LS is applied.

We finally design a Hybrid Memetic Algorithm (HMA) which combines the beneficial effect of the HGA and the MA for the achievement of improved node deployments.

The results show that the introduction of an enhanced combination of GA operators in the HGA enables the finding of better candidate solutions to the NLP in NLOS environments. Additionally, the introduction of knowledge in the optimization evolutionary (MA) process increases the overall performance in the solution of the NLP in a greater extent than the GA operators in the HGA. Finally, the HMA outperforms the previous configurations through the beneficial effect of the GA operators and the LS strategy of the MA. The HMA methodology proposed reaches an increase in accuracy in the optimization process in the scenario of simulations of this article of 14.2% with regards to previous GA optimizations of the literature. 

## Figures and Tables

**Table 1:** Asynchronous Time Difference of Arrival (A-TDOA) parameter configuration for the simulations, whose selection is based on [64,65].

Parameter	Magnitude
Frequency of emission	1090 MHz
Transmission power	400 W
Mean noise power	−94 dBm
Receptor sensibility	−90 dBm
Bandwidth	100 MHz
Clock frequency	1 GHz
Frequency–drift	U {−15,15} ppm
Time–frequency product	1
LOS path loss exponent	3.1
NLOS path loss exponent	4.5

**Table 2:** GA hyperparameters selected [30]. The resulting number of possible combinations *P*, obtained from Equation (2), shows the magnitude of the solution environment for the scenario proposed. Due to the number of possible solutions, the application of heuristic methodologies is in order.

GA Hyperparameters	Value
Population size	160
Convergence criteria	160 Generations or
	80% population equal
Elitism	18 %
Mutation	3%
Selection Technique	Tournament 2
Crossover Technique	Single-point
$RMSE_{ref}$	50 m
TLE points analyzed	1500
NLE points analyzed	24,000
Number of sensors *N*	5/8/11/14
Number of possible combinations *P*	$7.95\times 10^{21}/1.09\times 10^{35}/1.51\times 10^{48}/2.09\times 10^{61}$

**Table 3:** Comparison of multiple node distributions for the scenario proposed. Results displayed refer to the maximum, mean and minimum values of the Root Mean Squared Error (RMSE) array result, obtained from Equation (13), of a single distribution.

Node Distributions	Max RMSE (m)	Mean RMSE (m)	Min RMSE (m)
5 Nodes	23.02	5.32	0.63
8 Nodes	17.37	3.91	0.43
11 Nodes	10.28	2.31	0.29
14 Nodes	9.85	1.99	0.02

**Table 4:** Analysis of multiple combinations of genetic operators for the scenario proposed. Bold values shown are the minimum and mean values of the mean RMSE (m) of multiple simulation.

	Tournament 2		Tournament 3		Roulette	
	Min	Mean	Min	Mean	Min	Mean
**Single point**	2.31	2.669	2.547	2.67	2.66	2.9
**Two-point**	2.735	2.774	2.895	2.966	2.527	2.76
**Three-point**	**2.224**	**2.431**	2.826	2.998	**2.354**	**2.575**
**Uniform**	2.696	3.175	4.649	5.824	2.821	2.874

**Table 5:** RMSE comparison between the different methodologies analyzed. From the results obtained by the executed simulations, Bold Min RMSE refers to the mean RMSE value of the simulation that is the lesser of all simulations executed, whereas Mean RMSE refers to the mean value of mean RMSE of every simulation.

	Min RMSE (m)	Mean RMSE (m)
**GA-T2/MP3**	2.224	2.431
**GA-R/MP3**	2.354	2.575
**HGA**	**2.163**	**2.294**

**Table 6:** MA optimization results. Values displayed are the mean and minimal values of the mean RMSE of the simulations executed.

Node Distributions	Min RMSE (m)	Mean RMSE (m)
5 Nodes	4.287	4.923
8 Nodes	3.142	3.208
11 Nodes	2.184	2.284

**Table 7:** Comparison of positioning accuracy for each methodology studied in this paper for 8 nodes. Bold results displayed refer to the mean and minimal mean RMSE of every simulation executed.

Methodology	Min RMSE (m)	Mean RMSE(m)
GA	3.54	3.91
HGA	3.243	3.423
MA	3.142	3.208
HMA	**3.037**	**3.101**

## Abbreviations

The following abbreviations are used in this paper:

AGVs	Automatic Ground Vehicles
CRLB	Cramer–Rao Lower Bound
CS	Coordinator Sensor
FIM	Fisher Information Matrix
GA	Genetic Algorithm
GNSS	Global Navigation Satellite Systems
HGA	Hybrid Genetic Algorithm
HMA	Hybrid Memetic Algorithm
LOS	Line-of-Sight
LPS	Local Positioning Systems
LSD	Local Search Depth
MA	Memetic Algorithm
MP2	Multipoint Crossover 2
MP3	Multipoint Crossover 3
NLE	Node Location Environment
NLOS	Non-Line-of-Sight
NLP	Node Location Problem
R	Roulette Selection
T2	Tournament 2
T3	Tournament 3
TBP	Time-Based Positioning
TDOA	Time Difference of Arrival
TLE	Target Location Environment
TOA	Time Of Arrival
TS	Target Sensor
UAVs	Unmanned Aerial Vehicles
VND	Variable Neighborhood-Descent
WS	Worker Sensor
WSN	Wireless Sensor Network
